# Unraveling the impact of crizotinib to promote megakaryopoiesis for alleviating thrombocytopenia in myelodysplastic neoplasms

**DOI:** 10.1038/s41375-025-02729-w

**Published:** 2025-08-14

**Authors:** Hiroki Kobayashi, Yuta Komizo, Nanami Watanabe, Yu Miyata, Yoshiya Ohnuma, Yasushige Kamimura-Aoyagi, Kanako Yuki, Yoshihiro Hayashi, Minoru Yoshida, Yuka Harada, Hironori Harada

**Affiliations:** 1 Laboratory of Oncology, Tokyo University of Pharmacy and Life Sciences, Tokyo, Japan; 2 Laboratory of Cancer Pathobiology and Therapeutics, Ritsumeikan University, Kusatsu, Japan; 3 Chemical Genomics Research Group, RIKEN Center for Sustainable Resource Science, Wako, Japan; 4 Department of Clinical Laboratory, Tokyo Metropolitan Cancer and Infectious Diseases Center Komagome Hospital, Tokyo, Japan; 5 Hematology Division, Tokyo Metropolitan Cancer and Infectious Diseases Center Komagome Hospital, Tokyo, Japan; 6 Present Address: Drug Discovery Seeds Development Unit, RIKEN Center for Sustainable Resource Science, Wako, Japan

**Keywords:** Myelodysplastic syndrome, Haematological cancer

## Abstract

Current therapeutic options for myelodysplastic neoplasms (MDS)-associated thrombocytopenia are limited. Megakaryocyte maturation might be an innovative therapeutic strategy because its dysregulation profoundly contributes to MDS pathogenesis. Here, we identified crizotinib, a clinically approved anti-cancer drug for anaplastic lymphoma kinase (ALK)-positive non-small-cell lung cancer, as a potent inducer of megakaryocyte maturation. We demonstrated that crizotinib effectively induced polyploidization to increase the platelet-producing capacity of megakaryocytes derived from an MDS murine model and MDS patients by targeting Aurora kinases rather than its canonical targets, ALK/ROS1/c-MET. Importantly, crizotinib administration substantially ameliorated thrombocytopenia in our preclinical model. Our findings underscore the remarkable potential of crizotinib for drug repurposing and offer a novel therapeutic strategy for MDS patients with thrombocytopenia facing health-related quality of life concerns.

## Introduction

Myelodysplastic neoplasms (MDS) represent a heterogeneous group of clonal hematological disorders characterized by ineffective hematopoiesis and dysplastic changes in hematopoietic cells. Among the myriad complications associated with MDS, thrombocytopenia is a significant clinical challenge. Platelet transfusions are a common necessity for patients with severe thrombocytopenia; however, they provide only temporary relief and are associated with various risks, including alloimmunization and platelet refractoriness. Additionally, thrombopoietin (TPO) receptor agonists such as romiplostim [1, 2] and eltrombopag [3, 4] can be used in patients with low-risk MDS to stimulate platelet production by increasing the number of platelet precursors (i.e., megakaryocytes [MKs]) in the bone marrow (BM). However, there are infrequent but serious cases of progression to acute myeloid leukemia (AML) and BM fibrosis, possibly related to the expansion of abnormal hematopoietic stem cells [1, 5]; TPO has pivotal roles in megakaryopoiesis as well as the expansion of hematopoietic stem and progenitor cells (HSPCs). Notably, a recent clinical trial demonstrated that luspatercept, which promotes erythroid maturation by means of late-stage erythroblast differentiation, might potentially supersede erythropoiesis-stimulating agents (ESAs) as a first-line treatment for patients with anemia with lower-risk MDS who depend on red blood cell transfusions [6], underscoring the potential utility of a lineage maturation inducer for the treatment of matched-lineage cytopenia in MDS. Therefore, there is an emerging need for more specific anti-thrombocytopenic interventions that target megakaryopoiesis, as a counterpart to luspatercept.

Megakaryopoiesis includes an essential process called endomitosis that involves repeated rounds of DNA replication without cell division, resulting in MKs with polyploid nuclei. This unique adaptation enhances the capacity of MKs to produce platelets by increasing their cytoplasmic content and enabling the generation of a greater number of platelet-forming proplatelets [7]. However, dysregulated endomitosis might contribute to the pathogenesis of thrombocytopenia in MDS, as evidenced by observations of micromegakaryocytes and MKs with non-lobed nuclei in clinical studies [8, 9]. Therefore, understanding the mechanisms underlying endomitosis and its dysregulation holds promise for novel therapeutic interventions aimed at improving platelet production in MDS patients. Here, we address these issues using a chemical biology approach.

## Methods

### Study design

The objective of this study was to identify small molecules that promote megakaryopoiesis and evaluate their efficacy in ameliorating thrombocytopenia in MDS, using the mouse model and clinical samples. Imaging experiments using ex vivo cultured HPSCs from a model mouse were performed to identify compounds that promote megakaryopoiesis. After the identification of crizotinib, functional analyses, chemical profiling, and biochemical assays were performed to characterize its mode of action. To assess the clinical relevance of the mode of action of crizotinib, transcriptome analyses using CD34^+^ cells from MDS patients and HD were performed. All clinical samples used in this study were approved by the Institutional Research Ethics Committee of each participating research institution and were conducted in accordance with the Declaration of Helsinki. All the participants provided written informed consent. All animal experiments were approved and conducted in accordance with the Institutional Animal Care Protocol and Guidelines of the Tokyo University of Pharmacy and Life Sciences. Sample sizes and animal numbers for each experiment were not determined by power analysis but rather were chosen based on previous experience with similar setups that showed significance. The mice were randomly assigned to each study group. The investigators were not blinded to the experiments because drug administration required accurate dosing and monitoring of the animals’ responses to ensure proper treatment delivery and safety. No data were excluded.

### Clinical samples

Patients with MDS (*n* = 58) and healthy donor (HD, *n* = 8), who were donors for BM transplantation were enrolled in this study. All the participants provided written informed consent. This study received approval from the Institutional Research Ethics Committee of Tokyo Metropolitan Cancer and Infectious Diseases Center Komagome Hospital (approval No. 2203, 2640) and the Institutional Research Ethics Committee of Tokyo University of Pharmacy and Life Sciences (approval No. 17-4, 21-5). The study was conducted in accordance with the Declaration of Helsinki. Mononuclear cells (MNCs) were isolated from the BM aspirates of patients using Lymphoprep (Serumwerk Bernburg AG, Bernburg, Germany). MNCs were further processed for RNA-seq and MK differentiation assays (see below). The experiments using clinical samples are summarized in Supplementary Table 4.

### RNA-seq data analyses

CD34^+^ cells in the MNCs were purified using the EasySep Human CD34 Positive Selection Kit II (STEMCELL Technologies, Vancouver, Canada). Total RNA was isolated using an RNeasy Micro Kit (Qiagen, Hilden, Germany). Library preparation and sequencing were performed by Rhelixa Co., Ltd (Tokyo, Japan). Briefly, RNA-seq libraries were prepared using an NEBNext Poly(A) mRNA Magnetic Isolation Module (New England BioLabs, Beverly, USA) and an NEBNext Ultra II Directional RNA Library Prep Kit (New England BioLabs). Purified libraries were sequenced on an Illumina NovaSeq 6000, with 150 × 2 paired-end reads. FASTQ files were processed using AltAnalyze (version 2.1.0) [10]. Estimates of transcripts per million and reads per kilobase per million mapped reads were calculated using AltAnalyze *via* a built-in call to Kallisto for read pseudo-alignment and transcript quantification (EnMart72, hg19). Expression data were further analyzed using GSEA (see below) and hierarchical clustering. Hierarchical clustering was performed by Heatmapper [11] using the E2F targets and TNFα signals gene sets provided in Supplementary Table 3. The following parameters were used: average linkage (clustering method), Kendall’s Tau (distance measurement method), and application of clustering to rows and columns. The RNA-seq data obtained from clinical samples have been deposited in the Japanese Genotype-phenotype Archive affiliated with the DNA Data Bank of Japan under the accession number JGAS000724.

### GSEA

Hallmark (h.all.v2023.2.Hs.symbols.gmt) and Reactome (c2.cp.reactome.v2023.2.Hs.symbols.gmt) gene sets were tested for enrichment in our MDS cohort using GSEA (v4.1.0) [12, 13]. The following parameters were used for gene sorting: 1000 gene set permutations, Signal2Noise ranking metric, and descending-order real mode. For the analysis of public data (GSE114922), Hallmark plus E2F targets and TNFα signals (Supplementary Table 3) were tested for enrichment in MDS vs. HD using GSEApy (v1.1.2). The following parameters were used for gene sorting: 1000 gene set permutations, signal-to-noise ranking metric, and descending order.

### MK differentiation assay using human clinical samples

MNCs prepared from BM aspirates of patients were cultured in StemSpan SFEM II (STEMCELL Technologies) supplemented with StemSpan Megakaryocyte Expansion Supplement (STEMCELL Technologies) for 9–10 days. During the culture, the medium was replaced with fresh medium every two days. On days 9 and 10, the cells were treated with 0.1% DMSO or 1 μM crizotinib for three days. After further incubation for 24 h in drug-free media, the cells were fixed with ice-cold 70% ethanol. The fixed cells were washed once with FACS buffer [PBS containing 2% fetal calf serum (FCS; Nichirei, Tokyo, Japan) and 2 mM EDTA], and were stained with anti-CD41/CD61 antibody (A2A9/6, cat. No. 359808; BioLegend, San Diego, USA) for 30 min. The stained cells were washed once with FACS buffer, incubated for 10 min in FACS buffer containing 50 µg/mL propidium iodide (Nacalai Tesque, Kyoto, Japan) and 10 µg/mL RNase (Nippon Gene, Tokyo, Japan), and subjected to flow cytometry.

### Mice

C57BL/6 mice (7–10-week-old, female) were purchased from Tokyo Laboratory Animals Science (Tokyo, Japan). All animal studies were approved and conducted according to the Institutional Animal Care Protocol and Guidelines of Tokyo University of Pharmacy and Life Sciences (approval No. L20-07, L21-14, L22-08, L23-04, L24-12, and L25-25).

### Plasmids

To construct pMYs-*RUNX1*^S291fs^-IRES-GFP-2A-*CBL*^ΔE8/9^, T2A and *CBL*^ΔE8/9^ were PCR-amplified from pQCXIP-copGFP-2A-myc-His [14] and pMYs-*CBL*^ΔE8/9^-IRES-GFP [15], respectively. T2A and *CBL*^ΔE8/9^ were cloned into pMYs-*RUNX1*^S291fs^-IRES-GFP to generate pMYs-*RUNX1*^S291fs^-IRES-GFP-2A-*CBL*^ΔE8/9^. The constructed plasmids were verified by sequencing at FASMAC Co., Ltd. (Atsugi, Japan).

### Cell lines

Plat-E cells (RRID:CVCL_B488) were kindly provided by Dr. T. Kitamura (The University of Tokyo, Japan). HeLa cells (RRID:CVCL_0030) were kindly provided by Dr. Y. Maemoto (Tokyo University of Pharmacy and Life Sciences). Plat-E and HeLa cells were cultured in DMEM containing 10% FCS and 1% PS (FUJIFILM Wako, Osaka, Japan). Cell lines used in this study were not authenticated in our laboratory. No mycoplasma contamination was detected.

### Compounds

Crizotinib was purchased from Sigma-Aldrich (St. Louis, USA), MedChemExpress (Monmouth Junction, USA), and Combi-Blocks (San Diego, USA). Volasertib was purchased from Selleck (Munich, Germany). (*S*)-Crizotinib, alisertib, barasertib, BI2536, capmatinib, lorlatinib, and alectinib were purchased from MedChemExpress. BMS-5 and centrinone were purchased from Selleck. Capmatinib, lorlatinib, and alectinib were used at concentrations of 1 µM to inhibit their targets [16–18].

### Retrovirus production

Plat-E packaging cells were transfected with retrovirus vectors (pMYs-IRES-GFP, pMYs-IRES-hNGFR, pMYs-*RUNX1*^S291fs^-IRES-hNGFR, pMYs-*CBL*^ΔE8/9^-IRES-GFP, pMYs-*RUNX1*^S291fs^-IRES-GFP-2A-*CBL*^ΔE8/9^) using FuGENE HD (Promega, Madison, USA). After 24 h of incubation, the cells were fed fresh medium. Supernatants containing retroviruses were collected 48 h after transfection and filtered before transduction.

### Generation of transduced HSPCs and MDS mice

C57BL/6 donor mice (7–8-week-old, female) were euthanized *via* cervical dislocation under anesthesia. BM cells were isolated immediately from the femurs and tibias of the donor mice. BM HSPCs were then separated using the EasySep Mouse CD117 (c-Kit) Positive Selection Kit (STEMCELL Technologies). After stimulation with 50 ng/mL mouse stem cell factor (SCF, BioLegend), mouse FMS-like tyrosine kinase 3 ligand (BioLegend), mouse IL-6 (BioLegend), and mouse TPO (BioLegend), the cells were transduced with retrovirus(es) for 48 h using RetroNectin (Takara, Kusatsu, Japan)-coated dishes. Transduced HSPCs were obtained by sorting for the GFP expression in vitro. To generate MDS mice, non-sorted cells (including 5–10 × 10^5^ *CBL*^ΔE8/9^/*RUNX1*^S291fs^ double-transduced cells) were injected through the tail vein into sublethally irradiated wild-type recipient mice. For treatment of mice with crizotinib, 0.5% methylcellulose and 0.5% Tween-80 in water were used as vehicle.

### Complete blood counts

Blood counts were measured using pocH-100iV Diff (Sysmex, Kobe, Japan).

### Flow cytometric phenotyping

Flow cytometric data obtained using FACSCelesta (BD Biosciences, Franklin Lakes, USA) and CytoFLEX (Beckman Coulter, Brea, USA) were analyzed using FlowJo (v10.8.0, BD Biosciences). For MK size analysis, murine BM cells were stained with anti-CD41 (MWReg30, cat. No. 133916) and CD45 (30-F11, cat. No. 103106) antibodies (BioLegend). For murine BM/spleen MK ploidy analysis, cells fixed with ice-cold 70% ethanol were stained with anti-CD41 antibody (REA1194, cat. No. 130-122-767; Miltenyi Biotec, Bergisch Gladbach, Germany) or anti-CD41 antibody (MWReg30, cat. No. 133905; BioLegend), and 1 µg/mL DAPI (Nacalai Tesque). For megakaryocyte progenitor (MKP) detection, murine BM cells were stained with biotin-conjugated anti-mouse CD3e (145-2C11, cat. No. 100304), B220 (RA3-6B2, cat. No. 103204), Ter119 (Ter-119, cat. No. 116204), CD11b (M1/70, cat. No. 101204), and Gr-1 (RB6-8C5, 108404) antibodies (BioLegend) followed by staining with streptavidin-PerCP/Cy5.5 (BioLegend, cat. No. 405214) for lineage exclusion and with anti–c-Kit (2B8, cat. No. 105826), Sca1 (D7, cat. No. 108108), CD150 (TC15-12F12.2, cat. No. 115914), and CD41 (MWReg30, cat. No. 133914) antibodies (BioLegend). For megakaryocyte-erythroid progenitor (MEP) detection, the same antibody panel was used except that anti-CD150 and CD41 antibodies were replaced with anti-CD16/32 (93, cat. No. 101318; BioLegend) and CD34 (RAM34, cat. No. 562608; BD Biosciences) antibodies. Cells derived from transduced HSPCs were detected by staining with anti-human CD271 (ME20.4, cat. No. 345108; BioLegend) antibody and GFP expression.

### Cytospin and Diff-Quik staining

Cells were spun onto Matsunami SUPERFROST slides (Matsunami Glass, Osaka, Japan) for 5 min at 300 rpm using a Cyto-Tek 2500 cytocentrifuge (Sakura Finetek, Torrance, USA). The cells were then fixed and stained using Diff-Quik (Sysmex).

### Histological analyses and immunohistochemistry of paraffin-embedded bone marrow sections

Collected tibiae were fixed using 10% Formalin Neutral Buffer Solution (Fujifilm Wako). The specimens were decalcified, paraffin-embedded, and stained with hematoxylin and eosin at Genostaff Co., Ltd. (Tokyo, Japan). Histological sections were imaged and analyzed using BZ-X710 (Keyence, Osaka, Japan) and ImageJ (NIH) [19], respectively. For immunohistochemistry, paraffin sections were deparaffinized in xylene, rehydrated through a graded ethanol series, and permeabilized with 0.2% Triton X-100 in PBS for 2 minutes. Antigen retrieval was performed using Antigen Unmasking Solution, Citric Acid Based (Vector Laboratories, Newark, USA). After cooling, sections were blocked with 5% serum and 0.3% Triton X-100 in PBS for 1 hour at room temperature. Primary antibody incubation was carried out for 2 hours at room temperature using anti-von Willebrand factor (vWF) antibody (EPR25069-131, cat. No. ab287962; Abcam, Cambridge, UK). After washing three times with PBS, the sections were incubated with Alexa Fluor 594-conjugated goat anti-rabbit IgG antibody (Thermo, Waltham, USA; cat. No. A11012). Slides were mounted using ProLong Diamond Antifade Mountant (Thermo, cat. No. P36966). Images were acquired using an FV3000 confocal laser scanning microscope (Evident, Tokyo, Japan), and MK sizes were analyzed using ImageJ.

### Ex vivo culture of HSPCs

HSPCs were cultured in Ham’s F12 medium (FUJIFILM Wako) supplemented with 1% ITSX (Thermo), 10 mM HEPES, 1% PS, 100 ng/mL mouse TPO, 10 ng/mL mouse SCF, and 0.1% polyvinyl alcohol [a gift from Dr. Satoshi Yamazaki (The University of Tokyo, Japan)].

### Differentiation and ploidy analyses of cultured HSPCs

Cells were treated with or without the test compounds for 48 h. The cells were fed fresh medium, incubated for a further 48 h, and subjected to flow cytometry using anti-CD41 (MWReg30, cat. No. 133915; BioLegend) and anti-CD42d (1C2, cat. No. 148505; BioLegend) antibodies for differentiation assay, or fixed with ice-cold 70% ethanol for ploidy analysis. The fixed cells were stained with anti-CD41 antibody (MWReg30, cat. No. 133914; BioLegend) and 50 µg/mL propidium iodide (Nacalai Tesque) in the presence of 10 µg/mL RNase (Nippon Gene). The stained cells were subjected to flow cytometry. Ploidy was assessed by detecting ≥8 N MK or mean ploidy. The mean ploidy was calculated using the following formula: (2 N ×number of cells at 2 N ploidy level + 4 N ×number of cells at 4 N ploidy level +…+ 64 N ×number of cells at 64 N ploidy level/the total number of cells).

### Well plate imaging experiments

For drug testing, HSPCs derived from an MDS mouse were seeded on a U-bottom 96-well suspension culture plate (Greiner, Kremsmünster, Austria) at a density of 5000 cells/well. The cells were treated with 4 µM of each compound from Cambridge Cancer Compounds library (230 compounds, Selleck) or StemSelect Small Molecule Regulators (303 compounds, Millipore, Burlington, USA) for four days and imaged using a BZ-X810 microscope. Regarding the selection of bioactive compounds, a positive hit was defined as a condition in which compound treatment led to the emergence of morphologically large cells, with their proportion being equal to or greater than that observed under reference control conditions (HSPCs from a control mouse). For comparison of the phenotypic characteristics of HSPCs derived from control and MDS mice, cells were seeded on a U-bottom 96-well suspension culture plate at 5000 cells/well. After seeding for three days, the cells were imaged using a BZ-X710 microscope. For comparison of the phenotypic differences between control vector-transduced HSPCs and *CBL*^ΔE8/9^/*RUNX1*^S291fs^-transduced HSPCs, cells were seeded on a U-bottom 96-well suspension culture plate at a density of 5000 cells/well. After seeding for three days, the cells were imaged using a BZ-X710 microscope. For evaluation of the effects of crizotinib, HPSCs derived from an MDS mouse were seeded on a U-bottom 96-well suspension culture plate at a density of 5000 cells/well and treated with DMSO or crizotinib for 48 h. The cells were fed fresh medium, incubated for another 48 h, and imaged using a BZ-X710 microscope.

### Megakaryocyte colony-forming unit assay

The MegaCult-C Collagen and Medium with Lipids (StemCell Technologies) was used to assess megakaryocyte colony-forming units (CFU-MK). A combination of cytokines containing 50 ng/mL murine TPO (BioLegend), 10 ng/mL murine IL-3 (BioLegend), and 20 ng/mL human IL-6 (BioLegend) was used. HSPCs isolated from MDS mice were pretreated with either vehicle (DMSO) or 1 µM crizotinib for 48 h. The cells were then adjusted to 3.3 × 10^4^ cells/mL and cultured in collagen-based gel for 6 days before acetylcholinesterase staining. Colonies with three or more MKs were counted as MK colonies or as mixed MK colonies within a 150 mm^2^ area.

### Aurora A kinase assay in cultured cells

HeLa cells were treated with 100 ng/mL nocodazole for 24 h and then treated with the test compounds for 4 h. The cells were collected using trypsin, fixed with lyse/fix buffer (BD Biosciences) for 10 min, and permeabilized using Perm III buffer (BD Biosciences) for 30 min. After permeabilization, the cells were stained with Alexa Fluor 647-conjugated anti-phosphorylated T210 PLK1 (K50-483, cat. No. 558447; Millipore) and DAPI (Nacalai Tesque), and subjected to flow cytometry.

### Pharmacodynamic assay

C57BL/6 mice (10-week-old, female) were administered 100 mg/kg crizotinib or 30 mg/kg alisertib for 5 days qd. BM cells were collected from each mouse and fixed 2 h after the final administration using lyse/fix buffer (BD Biosciences) for 10 min. The fixed cells were permeabilized using Perm III buffer (BD Biosciences) for 30 min. The cells were stained with anti-Cyclin A2 (EPR17351, cat. No. ab181591; Abcam) or anti-pSer H3 (D2C8, cat. No. 3377; Cell Signaling, Danvers, USA) antibodies, followed by staining with Alexa Fluor 647-conjugated goat anti-rabbit IgG (Thermo, cat. No. A21244) and DAPI. The stained cells were subjected to intracellular flow cytometry.

### Gene expression analysis of transduced HSPCs treated with crizotinib

*CBL*^ΔE8/9^/*RUNX1*^S291fs^-transduced HSPCs were cultured in mTPO-containing medium (see above) and treated with DMSO or crizotinib for 48 h before collection. Total RNA was isolated from the cells using an RNeasy Mini Kit (Qiagen). Library preparation and sequencing were performed by Rhelixa Co., Ltd. Briefly, RNA-seq libraries were prepared using an NEBNext Poly(A) mRNA Magnetic Isolation Module (New England BioLabs) and an NEBNext Ultra II Directional RNA Library Prep Kit (New England BioLabs). Purified libraries were sequenced on an Illumina NovaSeq 6000, with 150 × 2 paired-end reads. FASTQ files were processed in AltAnalyze (version 2.1.0) [10]. Estimates of transcripts per million and reads per kilobase per million mapped reads were calculated using AltAnalyze *via* a built-in call to Kallisto for read pseudo-alignment and transcript quantification (EnsMart72, mm10). MK and platelet common marker genes were selected according to PanglaoDB [20]. The data were deposited in the Gene Expression Omnibus (GEO) under the accession number GSE267954.

### Proplatelet formation

Cells were plated on a 100 µg/mL fibrinogen-coated 96-well plate in HBSS supplemented with 10 ng/mL mouse IL-3 (BioLegend) and 30 µM S-nitrosoglutathione (FUJIFILM Wako). Fibrinogen was purchased from Sigma-Aldrich. After 3 h of incubation, the cells were fixed with 2% PFA for 15 min. After washing with PBS, the cells were stained with anti-beta I tubulin antibody (EPR16778, cat. No. ab179511; Abcam), followed by staining with Alexa Fluor 594-conjugated goat anti-rabbit IgG antibody (Thermo, cat. No. A11012). Nuclei were stained with Hoechst 33342 (Nacalai Tesque). After washing with PBS, the stained samples were observed under a confocal microscope and imaged (FV3000). The extent of proplatelet formation was quantified by categorizing cells into three groups based on protrusion morphology: “Intense” (cells with multiple and/or extensive protrusions exceeding 10 µm), “Weak” (cells with approximately two protrusions of around 10 µm in length), and “Minimal to No” (cells with fewer or shorter protrusions than the “Weak” category).

### Statistical analyses

The number of samples, animals, biological replicates, statistical tests, and significance are specified in each figure legend. In most figures, individual data points are shown to illustrate within-group distribution. Depending on the context, data are presented as mean ± standard deviation (s.d.), box plots with interquartile ranges, or violin plots to convey both the central tendency and the distribution shape. Data were assumed to follow a normal distribution and equal variances based on sample characteristics and prior experience with similar experimental settings. When either the assumption of normality or homogeneity of variance could not be confidently made, we employed Welch’s two-tailed *t*-tests, which remain robust under such violations. In cases involving repeated measures from the same subjects, two-tailed paired *t*-tests were used. For comparisons between independent groups, Student’s two-tailed *t*-tests were applied when the assumptions of normality and equal variance were considered valid. For analyses involving multiple group comparisons, one-way ANOVA with appropriate post-hoc corrections (Tukey’s or Dunnett’s test) was performed. Student’s *t*-tests, Welch’s *t*-tests, and paired *t*-tests were performed using Excel (for Mac 16.66; Microsoft). One-way ANOVA and post-hoc tests were conducted using R (v4.2.3).

## Results

### MDS mice exhibit thrombocytopenia mainly related to defects in MK maturation

**Fig. 1:**
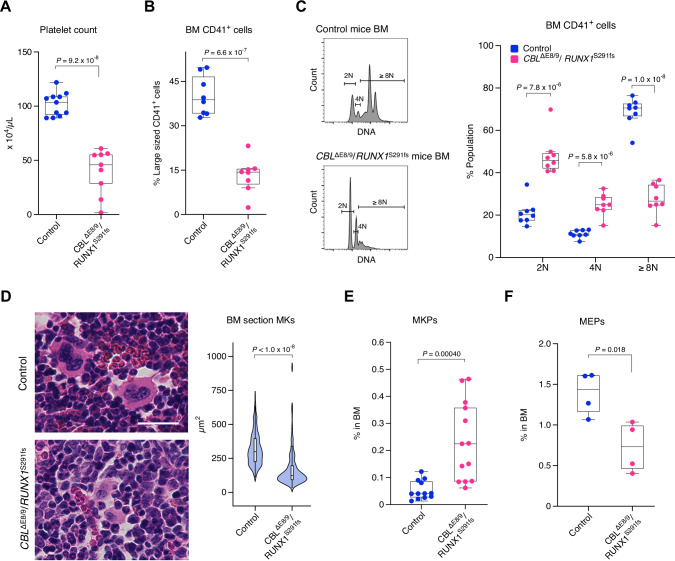
MDS mice exhibit thrombocytopenia mainly related to defects in MK maturation. **A** Platelet counts in peripheral blood from control mice (*n* = 11) and *CBL*^ΔE8/9^/*RUNX1*^S291fs^ mice (*n* = 9). **B** Sizes of CD41^+^ cells in BM from the indicated mice (*n* = 8). **C** Ploidy of CD41^+^ cells in BM from the indicated mice (*n* = 8). **D** Histological analysis of MKs in BM from the indicated mice. BM sections stained with hematoxylin and eosin were imaged (left) and MK size was quantified by measuring the area (right). More than 200 cells were evaluated from the mice (*n* = 4). Scale bar, 25 µm. **E** Population of MKPs in BM from control mice (*n* = 12) and *CBL*^ΔE8/9^/*RUNX1*^S291fs^ mice (*n* = 13). **F** Population of MEPs in BM from the indicated mice (*n* = 4). The gating strategies for **B**, **C**, **E**, and **F** were included in Supplementary Data 1. *P-*values were calculated using the Student’s two-tailed *t*-test (**A**–**C**, **E**, and **F**) and Welch’s two-tailed *t*-test (**D**).

We previously established a murine model of MDS using patient-derived *CBL* exon 8/9 deletion (*CBL*^ΔE8/9^) and *RUNX1* S291 frameshift mutation (*RUNX1*^S291fs^) *via* BM transplantation (BMT), which showed phenotypically relevant characteristics of MDS-ineffective hematopoiesis including thrombocytopenia [15]. In addition, we demonstrated that the pharmacological inhibition of Drp1 (Dynamin-related protein 1) attenuated leukocytopenia but not macrocytic anemia and the thrombocytopenia phenotypes. To address the mechanisms underlying the thrombocytopenia phenotype, we focused on the extent of MK maturation in MDS mice, because platelets are released from mature MKs. Ploidy and cell size reflect the extent of MK maturation. Flow cytometry and histological analyses demonstrated that MKs in the BM of MDS mice exhibited lower ploidy and reduced size compared to control mice, as reflected in the reduced platelet counts (Fig. 1A–D and Supplementary Fig. 1A), consistent with clinical observations [8, 9]. To further investigate the underlying defects in megakaryopoiesis, we examined MK progenitor populations, specifically unipotent megakaryocyte progenitors (MKPs) and bipotent megakaryocyte-erythroid progenitors (MEPs). We found that MKPs were expanded in the BM of MDS mice (Fig. 1E), while MEPs were reduced (Fig. 1F), consistent with previous findings regarding MEP depletion in MDS mice [15]. Given that MDS mice exhibited splenomegaly (Supplementary Fig. 1B), likely as a compensatory response to cytopenia, we also assessed MK maturation in the spleen. In control mice, splenic MKs were less mature than their BM counterparts, consistent with prior reports [21, 22]. In MDS mice, although a modest increase in the 4 N population was observed among splenic MKs compared to controls, no substantial changes were detected (Supplementary Fig. 1C). Since the BM is the principal site of platelet production in mice [23], these findings suggest that thrombocytopenia in MDS mice primarily results from defective differentiation and maturation of BM MKs, compounded by disruptions in stem cell architecture.

### Ex vivo culture of HSPCs from MDS mice reflects the disease phenotypes of MKs in MDS

**Fig. 2:**
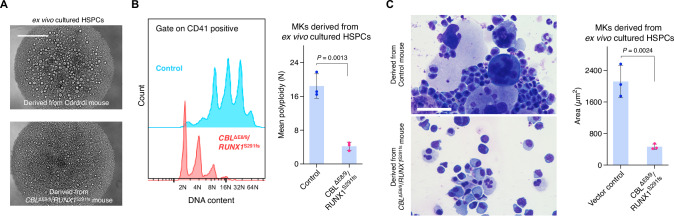
In vitro recapitulation of the disease phenotypes of MKs in MDS. The phenotypes of MKs derived from ex vivo cultured HSPCs from MDS model mice. **A** Representative images of ex vivo cultured HSPCs from the indicated mice. Large cells in the upper panel were matured MKs derived from HSPCs. Scale bar, 400 µm. **B** Ploidy of MKs derived from ex vivo cultured HSPCs from the indicated mice. The data represent the mean ± s.d. of three independent experiments. **C** Diff-quik staining of ex vivo cultured HSPCs from the indicated mice (left). Scale bar, 50 µm. MK size was quantified using ImageJ (right). The data represent the mean ± s.d. of three independent experiments with >5 fields of view per sample. The gating strategy for B was included in Supplementary Data 2. *P-*values were calculated using the Student’s two-tailed *t*-test (**B**, **C**).

Small molecules that promote MK maturation can attenuate the thrombocytopenia phenotype caused by maturation defects. To screen for such molecules using an in vitro assay system, we assessed the disease phenotype of HSPCs from MDS mice ex vivo. Consistent with the disease phenotypes in vivo, flow cytometric and imaging analyses revealed that HSPCs from MDS mice produced small MK progenitors with low ploidy compared with normal HSPCs in an ex vivo culture, even in the presence of TPO (Fig. 2A–C). We investigated whether these phenotypes would manifest as a result of mutant proteins produced after retroviral transduction into HSPCs to generate MDS mice. As shown in Supplementary Figure 2A–C, transduction of mutated genes into HSPCs was sufficient for the manifestation of defective phenotypes, suggesting that the phenotypes in vitro were cell-intrinsic. Notably, MKs derived from non-transduced HSPCs in MDS mice exhibited the small-size phenotype (Supplementary Fig. 3). While additional complexities are likely to exist in the in vivo environment, these findings suggest that the observed MK maturation defects are predominantly driven by gene mutations.

### Identification of crizotinib as an MK maturation inducer

**Fig. 3:**
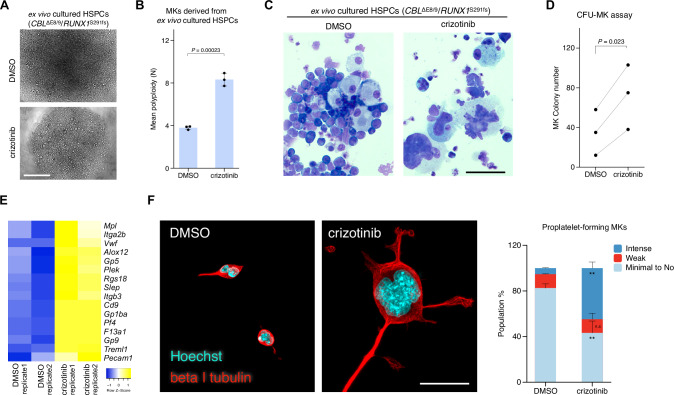
Crizotinib promotes MK maturation in vitro. Effects of crizotinib on phenotypes of MKs derived from ex vivo cultured HSPCs from MDS model mice and HSPCs transduced with *CBL*^ΔE8/9^/*RUNX1*^S291fs^. **A** Representative images of DMSO- or crizotinib-treated ex vivo cultured HSPCs from MDS mice. Scale bar, 400 µm. **B** Effect of crizotinib on the ploidy of MKs derived from ex vivo cultured HSPCs from MDS mice. The data represent the mean ± s.d. of three independent experiments. **C** Effects of crizotinib on the morphology of ex vivo cultured HSPCs from MDS mice. Scale bar, 50 µm. **D** The megakaryocyte colony-forming units (CFU-MK) in crizotinib-treated HSPCs from MDS mice. The data are from three independent mice. **E** Gene expression analysis of MK and platelet markers in *CBL*^ΔE8/9^/*RUNX1*^S291fs^–transduced HSPCs treated with DMSO or crizotinib for 48 h (*n* = 2, biological replicates). **F** Representative images of proplatelet formation in DMSO- or crizotinib- treated MKs derived from ex vivo cultured HSPCs from MDS mice (left; cyan, Hoechst 33342; red, beta I tubulin). Scale bar, 30 µm. Proplatelet-forming MKs were quantified across three independent experiments, analyzing more than 10 fields of view per condition (right). *P*-values were calculated using the paired two-tailed *t*-test (**D**) and the Student’s two-tailed *t*-test (**B**, **F**). *n.s*., not significant; **, *P* < 0.01.

To explore compounds that promote MK maturation, we performed imaging experiments using HSPCs from MDS mice and small-molecule collections. We identified five compounds that were likely to increase the cell size (Supplementary Fig. 4). Of the five compounds, we validated the effect of crizotinib, an approved drug for anaplastic lymphoma kinase (ALK)-rearranged non-small-cell lung cancer (NSCLC), because of its unreported bioactivity and potential for drug repurposing. Imaging and flow cytometric analyses revealed that crizotinib induced MK polyploidization and increased the cell size (Fig. 3A–C). In addition, crizotinib treatment significantly increased both the number of MK colonies and the CD41^+^ CD42d^+^ cell population, providing the evidence that crizotinib promotes MK differentiation (Fig. 3D and Supplementary Fig. 5A). The treatment of HSPC transductants with crizotinib also increased polyploidization and cell size (Supplementary Fig. 5B, C). Notably, the total number of cells obtained by the addition of crizotinib was approximately one-tenth of that observed with vehicle (dimethyl sulfoxide [DMSO]), reflecting the induction of differentiation. In addition to the phenotypic changes, crizotinib had an impact on the gene expression profiles; the treatment of HSPC transductants with crizotinib for 48 h increased the expression levels of MK and platelet-related genes (Fig. 3E). The effects of crizotinib on MK maturation have not been reported; therefore, we confirmed the bioactivity of crizotinib across chemical suppliers (Supplementary Fig. 6), ensuring that crizotinib promotes MK maturation. To determine whether crizotinib-induced maturation of MK was functional, we analyzed proplatelet formation and found that MKs increased in ploidy and size by crizotinib markedly formed proplatelets (Fig. 3F). Together, these results demonstrate that crizotinib induces the maturation of MKs, which are functional.

### Crizotinib promotes MK maturation mainly by targeting Aurora kinases

**Fig. 4:**
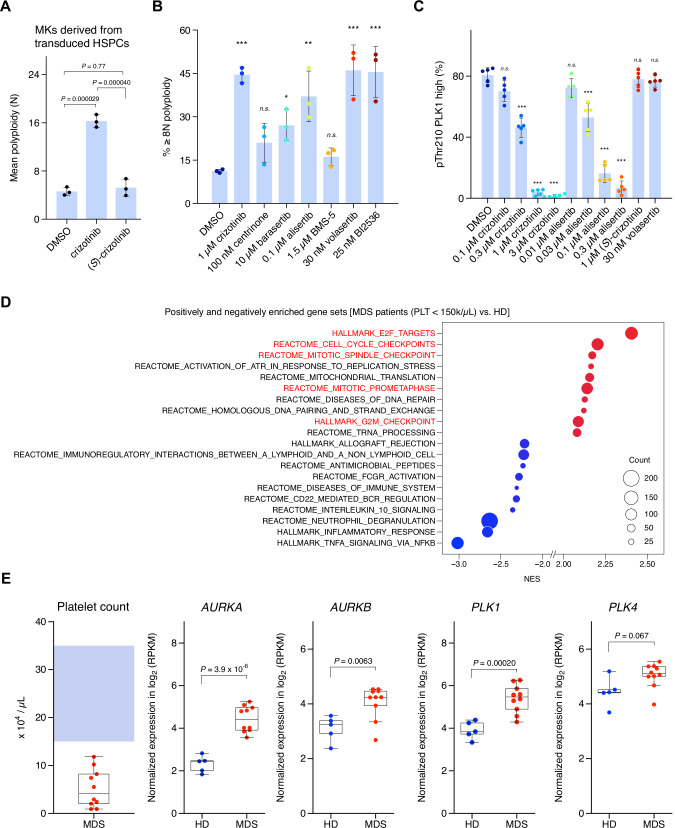
Crizotinib promotes MK maturation by inhibiting Aurora kinases, addressing the dysregulated cell cycling observed in MDS patients with thrombocytopenia. **A** Effect of (*S*)-crizotinib on the ploidy of MKs derived from *CBL*^ΔE8/9^/*RUNX1*^S291fs^–transduced HSPCs. The data represent the mean ± s.d. of three independent experiments. **B** Effects of inhibitors against PLK1, and kinases to which crizotinib binds, on the ploidy of MKs derived from *CBL*^ΔE8/9^/*RUNX1*^S291fs^–transduced HSPCs. The data represent the mean ± s.d. of three independent experiments. **C** Effects of the indicated inhibitors on the phosphorylation levels of PLK1 T210, evaluated by flow cytometric analysis. The data represent the mean ± s.d. of five independent experiments. **D** The top 10 gene sets positively or negatively enriched in BM CD34^+^ cells from MDS patients with thrombocytopenia [PLT (platelet count) < 15 × 10^4^/µL, *n* = 10)] vs. those from HD (*n* = 5) by GSEA (Hallmark & Reactome gene sets). Gene sets related to cell cycle progression are highlighted in red. NES, normalized enrichment score. The top 100 lists were included in Supplementary Tables 1 and 2. (**E**) Platelet counts of the MDS patients and relative gene expression levels of *AURKA*, *AURKB*, *PLK1*, and *PLK4* in cells analyzed in D. RPKM, reads per kilobase per million mapped reads. The blue-shaded area in the figure represents the normal range of platelet counts in healthy individuals. The gating strategy for C was included in Supplementary Data 3. *P-*values were calculated using one-way ANOVA with Tukey’s multiple comparison test [A (vs. DMSO), C (vs. DMSO)], one-way ANOVA with Dunnett’s multiple comparison test (**B**), and Welch’s two-tailed *t*-test (**E**). *n.s*., not significant; *, *P* < 0.05; **, *P* < 0.01; ***, *P* < 0.001.

Crizotinib is an ATP-competitive inhibitor targeting ALK/ROS1/c-MET kinases, which has been approved by the Food and Drug Administration (FDA), the European Medicines Agency (EMA), and the Pharmaceuticals and Medical Devices Agency (PMDA) as a first-in-class chemical for the treatment of NSCLC and other cancers with *ALK* rearrangement, *ROS1* rearrangement, or aberrant activation of c-MET [24–27]. Therefore, we examined whether inhibition of these molecules is involved in the induction of MK maturation. We used alectinib (an ALK inhibitor) [16], lorlatinib (an ALK/ROS1 inhibitor) [17], and capmatinib (a c-MET inhibitor) [18]. Neither a single treatment with each drug nor combined treatment with alectinib/capmatinib or lorlatinib/capmatinib induced MK polyploidization (Supplementary Fig. 7). This suggests that crizotinib induced MK polyploidization by inhibiting molecules other than ALK/ROS1/c-MET. To determine whether crizotinib induces MK polyplidization by inhibiting certain kinase(s), we evaluated the effect of (*S*)-crizotinib, an enantiomer of crizotinib that has lost its ability to bind to various kinases [28]. As shown in Fig. 4A, (*S*)-crizotinib failed to induce MK polyploidization, suggesting that MK polyploidization is induced by the inhibitory effect of crizotinib on certain kinase(s). Failure of cytokinesis by disrupting actomyosin contraction or cell cycle regulators in G_2_/M can lead to endomitosis and MK polyploidization [29]. The KINOMEscan study revealed kinases that bind to crizotinib [30], among which those implicated in cytokinesis, ranked by increasing dissociation constant (*K*_*d*_), include PLK4 (*PLK4*, Polo-like kinase 4), Aurora B kinase (*AURKB*), Aurora A kinase (*AURKA*), and LIMK1/2 (*LIMK1/2*, LIM kinase 1/2) (Supplementary Fig. 8). We therefore evaluated the effect of each selective inhibitor against the kinase on MK polyploidization. As a result, barasertib (an Aurora B kinase inhibitor) and alisertib (an Aurora A kinase inhibitor) induced MK polyploidization (Fig. 4B). Consistent with the KINOMEscan study, crizotinib inhibits the enzymatic activity of Aurora A/B kinases in vitro [31]. On the one hand, Aurora B kinase inhibitors can increase microtubule destruction to induce endomitosis through stathmin and mitotic centromere-associated kinesin action [32]. On the other hand, previous studies have demonstrated that alisertib or the conditional knockout of *Aurka* in the hematopoietic lineage induces MK polyploidization [33, 34]; however, the molecular mechanisms underlying the induction of polyploidization remain unclear. To address this, we surveyed substrates of Aurora A kinase, whose perturbation induces MK polyploidization. We focused on Polo-like kinase 1 (PLK1) as a candidate substrate; Aurora A kinase phosphorylates PLK1 T210, which in turn drives mitotic events [35, 36]. Indeed, volasertib and BI2536, selective PLK1 inhibitors, induced MK polyploidization (Fig. 4B). We confirmed that crizotinib inhibited Aurora A kinase in cultured cells, and the concentration range at which crizotinib decreased the phosphorylation level of PLK1 T210 corresponded with the range at which it promoted MK maturation, as was also observed for alisertib (Fig. 4C). Volasertib did not decrease the phosphorylation level of PLK1 T210 because volasertib does not inhibit Aurora A kinase, which is consistent with previous reports [37, 38]. These data demonstrated that crizotinib promotes MK polyploidization by inhibiting both Aurora B kinase and Aurora A kinase, with the suppression of PLK1 activity resulting from Aurora A kinase inhibition, thereby contributing to the endomitotic process. As mentioned above, endomitosis is characterized as a failure of cytokinesis. We therefore hypothesized that megakaryocyte progenitors in subsets of MDS patients with thrombocytopenia would elevate expression levels of mitotic genes, including *AURKA*, *AURKB*, and *PLK1*, which in turn would promote cytokinesis/cell cycling rather than endomitosis. To obtain evidence supporting this notion, we analyzed the transcriptomes of MDS patients, with a focus on the thrombocytopenia phenotype. Gene Set Enrichment Analysis (GSEA) revealed that gene sets related to cell cycle progression, especially mitosis (gene sets highlighted in red) including “AURKA_ACTIVATION_BY_TPX2,” “REGULATION_OF_PLK1_ACTIVITY_AT_G2_M_TRANSITION,” and “POLO_LIKE_KINASE_MEDIATED_EVENTS,” were positively enriched whereas inflammatory response genes were negatively enriched, in BM CD34^+^ cells from MDS patients with low platelet counts (<15 × 10^4^/µL, *n* = 10) vs. those from healthy donors (HD, *n* = 5) (Fig. 4D, Supplementary Tables 1, 2). Indeed, the expression levels of *AURKA*, *AURKB*, and *PLK1* but not *PLK4* were higher in patients than in HD (Fig. 4E). Hierarchical clustering analysis demonstrated that the expression profiles of the positively and negatively enriched genes (Supplementary Table 3; E2F targets and TNFα signals, respectively) in the analysis tended to be observed in the subset of MDS patients associated with thrombocytopenia rather than in HD and other patients with normal platelet counts (Supplementary Fig. 9A and Supplementary Table 4). E2F targets include *AURKA*, *AURKB*, and *PLK1*, and their high expression is characterized as a cell cycling signature. The HD cluster was composed of a population with very low (E2F targets)　and very high (TNFα signals) expressions; cluster I was composed of those with high (E2F targets) and low (TNFα signals) expressions; cluster IIa was composed of those with very high (E2F targets) and very low (TNFα signals) expressions; cluster IIb was composed of those with very high (E2F targets) and low (TNFα signals) expressions; cluster III was composed of those with low (E2F targets) and high (TNFα signals) expressions; and cluster IV was composed of those with very low (E2F targets) and low (TNFα signals) expressions. Although clusters IIb and IV mainly contained MDS patients with thrombocytopenia, the blast status was different between clusters; MDS with increased blasts (MDS-IB) patients were enriched in cluster IV, whereas MDS–non-IB patients were enriched in cluster IIb. This indicates that dysregulation of the cell cycling program is associated with thrombocytopenia in MDS–non-IB patients. Consistently, the analysis of a GSE114922 public dataset [39] revealed that E2F targets, including PLK1, were expressed at a higher level in BM CD34^+^ cells from MDS–non-IB and MDS with ringed sideroblasts (MDS–RS) patients than in those from MDS-IB patients and HD (Supplementary Fig. 9B, C, and Supplementary Table 5). These findings support the notion that dysregulation of the cell cycling program can advance cytokinesis rather than endomitosis in MDS (especially unassociated with blasts), which in turn leads to thrombocytopenia.

### Crizotinib promotes MK maturation in vivo and increases platelet counts

**Fig. 5:**
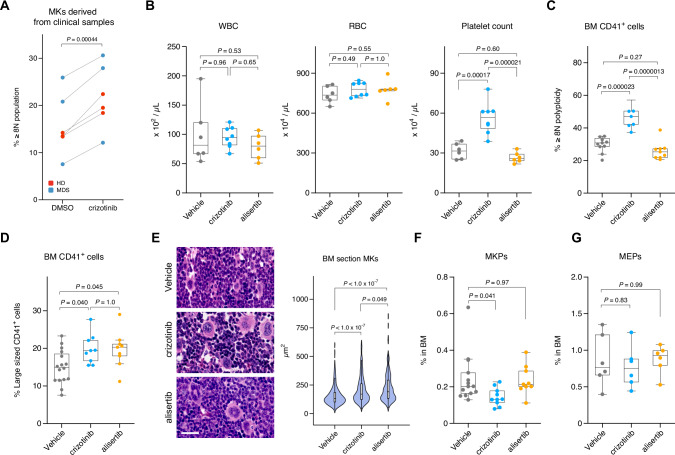
Crizotinib promotes MK maturation in clinical human samples and MDS mice leading to increased platelet counts. **A** Effect of crizotinib on the polyploidy of MKs derived from clinical human samples (*n* = 6). **B** Effects of vehicle (*n* = 6), crizotinib (*n* = 8), and alisertib (*n* = 6) on complete blood counts in MDS mice. WBC, white blood cell. RBC, red blood cell. **C** Flow cytometric analysis of ploidy of MKs in the BM of MDS mice administered vehicle (*n* = 10), crizotinib (*n* = 6), or alisertib (*n* = 9). **D** Flow cytometric analysis of the size of MKs in the BM of MDS mice administered vehicle (*n* = 16), crizotinib (*n* = 9), or alisertib (*n* = 9). **E** Histological analysis of hematoxylin and eosin-stained BM sections from MDS mice administered vehicle, crizotinib, or alisertib. MK size was quantified using the obtained images. Scale bar, 25 µm. More than 350 cells were evaluated from the indicated mice (*n* = 6). **F** Population of MKPs in the BM from MDS mice administered vehicle (*n* = 14), crizotinib (*n* = 10), or alisertib (*n* = 9). **G** Population of MEPs in the BM from MDS mice administered vehicle, crizotinib, or alisertib (*n* = 6). *P*-values were calculated using the paired two-tailed *t*-test (A), one-way ANOVA with Tukey’s multiple comparison test (**B**–**E**), and one-way ANOVA with Dunnett’s multiple comparison test (**F**, **G**).

To test whether crizotinib promotes MK maturation in human cells, we used BM mononuclear cells derived from clinical samples. Crizotinib increased ploidy, even in cells derived from HD, suggesting that crizotinib can act on human cells independently of their DNA mutation status (Fig. 5A). To determine whether crizotinib affects the activity of Aurora A/B kinase in vivo, we performed a pharmacodynamic analysis of crizotinib and alisertib by detecting the G_2_- and/or M-phase population in BM cells from drug-treated mice by flow cytometry using anti-Cyclin A2 and anti–phospho-Ser10 histone H3 antibodies. Cyclin A2 expression is increased from the S to G_2_ phase, forming a complex with CDK2 to induce DNA replication. Cyclin A2 is subsequently degraded *via* the ubiquitin-dependent proteolytic pathway in the early M phase; therefore, the simultaneous measurement of Cyclin A2 and DNA content can easily distinguish between the M and S/G_2_ phases [40, 41]. The phosphorylation of H3 at Ser10, which is also used as an M phase marker, is mainly catalyzed by Aurora B kinase [42]. We found that crizotinib as well as alisertib increased the G_2_/M- and M-phase population in the BM by detecting the DNA content and Cyclin A2 expression. Alisertib, but not crizotinib, increased the population size with H3 Ser10 phosphorylation (Supplementary Fig. 10A, B). These data suggest that crizotinib inhibits Aurora A/B kinases in vivo, while alisertib selectively inhibits Aurora A kinase. To examine the potential of crizotinib for drug repurposing as a therapy against thrombocytopenia in MDS, we evaluated its efficacy in MDS mice in a side-by-side comparison with alisertib. MDS mice were administered crizotinib or alisertib (for 5 days qd, po, 2 days off × 4 cycles; started on day 17 after BMT). Crizotinib improved the platelet count without altering the counts of leukocytes or red blood cells (Fig. 5B). Flow cytometric analysis revealed that crizotinib increased the ploidy and size of MKs in the BM (Fig. 5C–E). In contrast to crizotinib, alisertib failed to improve the platelet count and ploidy of MKs (Fig. 5B, C) although the size of MKs was increased (Fig. 5D, E). A notable difference between crizotinib and alisertib was observed in the proportion of the MKP population; crizotinib but not alisertib reduced the MKP pool to be differentiated (Fig. 5F). Neither crizotinib nor alisertib showed any significant effect on the MEP population (Fig. 5G). These findings collectively demonstrate that crizotinib treatment improves platelet production, presumably by promoting the differentiation of MKPs to MKs and their maturation through the inhibition of Aurora kinases (Supplementary Fig. 11). Although further investigation of the therapeutic mechanisms related to the beneficial effects of crizotinib treatment is required, our data suggest that crizotinib has great potential as a therapeutic agent against thrombocytopenia in MDS.

## Discussion

The heterogeneity of MDS, characterized by diverse genetic aberrations and clinical presentations, demands a complex and personalized variety of therapeutic approaches. Currently, the treatment of MDS is generally based on an individual’s risk of progression to AML and estimated survival calculated based on the revised international prognostic scoring system [43]. The only curative management of very high, high, and possibly intermediate risk of disease is with allogeneic hematopoietic stem cell transplantation, which is accessible to only a small number of fit patients. The therapeutic options for the remaining high-risk MDS patients are hypomethylating agents, which promote clinical responses in a subset of patients. However, the management of anemia in low- and very low-risk MDS patients involves ESAs, granulocyte-macrophage colony-stimulating factor, lenalidomide for 5q- and other MDS, luspatercept, and hypomethylating agents [44]. In addition, immunosuppressive agents such as anti-thymocyte globulin and cyclosporine A have been used with some success in patients with low-risk MDS [45, 46]. In contrast, treatment options are severely restricted for patients with persistent thrombocytopenia who require platelet transfusions. While TPO receptor agonists have been evaluated in clinical trials for MDS patients, they are not yet approved for this indication. In this study, we propose an alternative therapeutic strategy targeting megakaryopoiesis for the management of thrombocytopenia in MDS. Our in vitro studies demonstrated that crizotinib promotes the differentiation of megakaryocyte precursors that are unresponsive to TPO stimulation, suggesting its potential efficacy for patients who are refractory to TPO receptor agonists.

Our phenotype-based chemical biology approach identified crizotinib and clarified the involvement of the dysregulation of the cell cycling program in MK maturation defects; the cell cycling signature (E2F targets) was found in cells from MDS–non-IB patients with thrombocytopenia across two independent clinical datasets. To the best of our knowledge, this is the first report providing insights into the mechanism underlying MK maturation defects in the context of thrombocytopenia in MDS. Although further investigation of the molecular mechanisms involved in thrombocytopenia associated with such a gene expression profile is required, responsive mediators might be *E2F-1* and *Myc*, which can be activated by E2F-1 [47]. *E2F-1* or *Myc-ER* overexpression under the control of a *PF4* promoter blocked MK differentiation and caused an accumulation of MK precursors and a decrease in the size and ploidy of MKs in vivo [48, 49]. Clinically, E2F-1 protein expression or *Myc* mRNA expression was higher in cells from MDS patients than in those from HD [50–52]. Consistent with these observations and in addition to E2F targets, Myc targets (HALLMARK_MYC_TARGETS_V1 and HALLMARK_MYC_TARGETS_V2) were enriched in MDS patients with thrombocytopenia and in MDS–non-IB patients in our clinical data (Supplementary Table 1) and the GSE114922 dataset (Supplementary Table 5), respectively. Notably, Myc facilitates the MYC–AURKA/PLK1 axis feed-forward circuit in lymphomas [53, 54] and neuroblastomas [55, 56]. Based on these findings, we consider MDS–non-IB patients as potential responders to crizotinib for therapy against thrombocytopenia.

A genetic study using conditional knockout of *Aurka* in hematopoietic lineages demonstrated that, while *Aurka* is indispensable for adult hematopoiesis, it is not required for MK differentiation and maturation [33]. In contrast, our findings revealed that crizotinib induces MK differentiation and maturation both in vitro and in vivo, leading to an increase in platelet counts in the preclinical model. The differential selectivity between crizotinib and alisertib for Aurora kinases suggests that crizotinib’s therapeutic effect may be mediated through either the pan-inhibition of Aurora kinases or the specific kinase inhibition profile. Notably, crizotinib exhibits lineage specificity; while promoting MK differentiation and platelet production, it does not affect leukocyte or red blood cell counts. In mice, this selectivity is likely due to the relatively short half-life of approximately five hours [57], allowing the transient inhibition of Aurora kinases and sparing other hematopoietic lineages from disruption. However, crizotinib exhibits a longer half-life of approximately 42 h in humans [58], which could alter its pharmacodynamic profile. This highlights the need to carefully optimize dosing regimens, in terms of both dose and frequency, for human applications to balance therapeutic efficacy and safety.

Although our preclinical studies provide compelling evidence for the efficacy of crizotinib in ameliorating thrombocytopenia in MDS, its clinical efficacy in increasing platelet counts remains unproven. To date, clinical studies have focused on healthy volunteers and cancer patients, where crizotinib has demonstrated an acceptable safety profile [59]. However, in healthy individuals, MK differentiation and maturation are not impaired, making it unlikely that crizotinib increases platelet counts in this context. In patients with solid cancers, disease progression often influences platelet levels, which complicates the assessment of crizotinib efficacy. To translate our findings into clinical practice, several critical steps must be taken. Investigator-initiated clinical trials are essential to confirm the efficacy of crizotinib in MDS patients with thrombocytopenia. Pharmacokinetic and pharmacodynamic studies are particularly important, given the differences in crizotinib half-life between mice and humans, to determine the optimal dosing strategies that achieve transient but effective Aurora kinase inhibition in humans. Furthermore, although MDS–non-IB patients are anticipated to be primary responders to crizotinib, comprehensive biomarker studies could help identify patient subgroups that are most likely to benefit from this therapy. By addressing these key challenges, future research can bridge the gap between preclinical promise and clinical application, potentially offering a novel therapeutic option for MDS patients with thrombocytopenia.

## Supplementary information

Supplementary information

Supplementary Table 1

Supplementary Table 2

Supplementary Table 3

Supplementary Table 4

Supplementary Table 5

## Data Availability

The plasmids constructed in this study are available from the authors, with a standard material transfer agreement with Tokyo University of Pharmacy and Life Sciences. KINOMEscan data in Supplementary Fig. 8 are publicly available at Library of Integrated Network-based Cellular Signatures (http://lincs.hms.harvard.edu/db/datasets/20200/). The RNA-seq data reported in Fig. 3E have been deposited in the GEO under the accession number GSE267954. The RNA-seq data analyzed in Supplementary Fig. 9B and 9C are publicly available in the GEO under the accession number GSE114922. The RNA-seq data obtained from clinical samples have been deposited to the Japanese Genotype-phenotype Archive affiliated with the DNA Data Bank of Japan under the accession number JGAS000724 (https://humandbs.dbcls.jp/en/hum0473-v1). All other data generated or analyzed during this study are included in the main text or the supplementary materials.
