# Supplementary material for: Unraveling the impact of crizotinib to promote megakaryopoiesis for alleviating thrombocytopenia in myelodysplastic neoplasms
Source: Leukemia. 2025 Aug 14;39(11):2789–800. doi: 10.1038/s41375-025-02729-w (PMC12589124; doi:10.1038/s41375-025-02729-w)
Supplement: Supplementary file 1 — Supplementary information [file 41375_2025_2729_MOESM1_ESM.pdf]

Supplementary Information for

Unraveling the impact of crizotinib to promote megakaryopoiesis for alleviating thrombocytopenia in myelodysplastic neoplasms

Hiroki Kobayashi, Yuta Komizo, Nanami Watanabe, Yu Miyata, Yoshiya Ohnuma, Yasushige Kamimura-Aoyagi, Kanako Yuki, Yoshihiro Hayashi, Minoru Yoshida, Yuka Harada, and Hironori Harada

Corresponding author: hkbys@toyaku.ac.jp

The PDF file includes:

Supplementary Figs. 1 to 11
Supplementary Data 1 to 4
Legends for Supplementary Tables 1 to 5

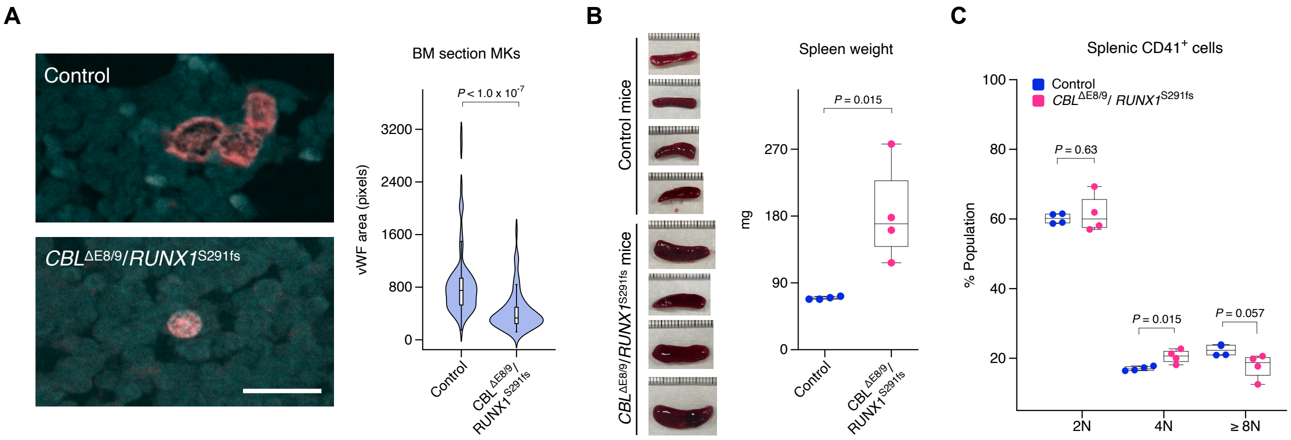

Supplementary Fig. 1

Analysis of MKs in BM and spleen from MDS mice. (**A**) Representative images of BM sections from the indicated mice immunostained with anti-von Willebrand factor (vWF) antibody (red) to visualize MKs and counter stained with DAPI (cyan) (left). Scale bar, 20 µm. Quantitative analysis of MK size was performed by measuring the cell area (right). Data represent analysis of >70 cells from four independent mice (n = 4). (**B**) Spleen size and weight from the indicated mice (n = 4). (**C**) Ploidy analysis of splenic $CD41^{+}$ cells from the indicated mice (n = 4). P-values were calculated using Welch's two-tailed t-test (A) and the Student's two-tailed t-test (B, C).

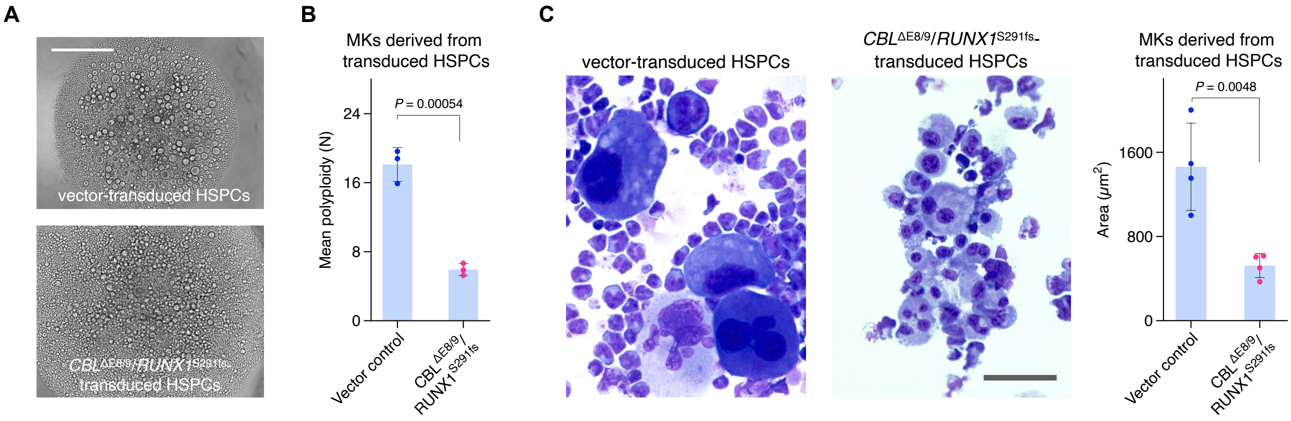

Supplementary Fig. 2

The disease phenotypes of MKs derived from HSPCs transduced with genes facilitating MDS. (**A**) Representative images of transduced HSPCs. Scale bar, 400 µm. (**B**) Ploidy of MKs derived from transduced HSPCs. The data represent the mean ± s.d. of three independent experiments. (**C**) Diff-quik staining of transduced HSPCs (left). Scale bar, 50 µm. MK size was quantified using ImageJ (right). The data represent the mean ± s.d. of four independent experiments with >5 fields of view per sample. The gating strategy for B was included in Supplementary Data 2. *P*-values were calculated using the Student's two-tailed *t*-test (B, C).

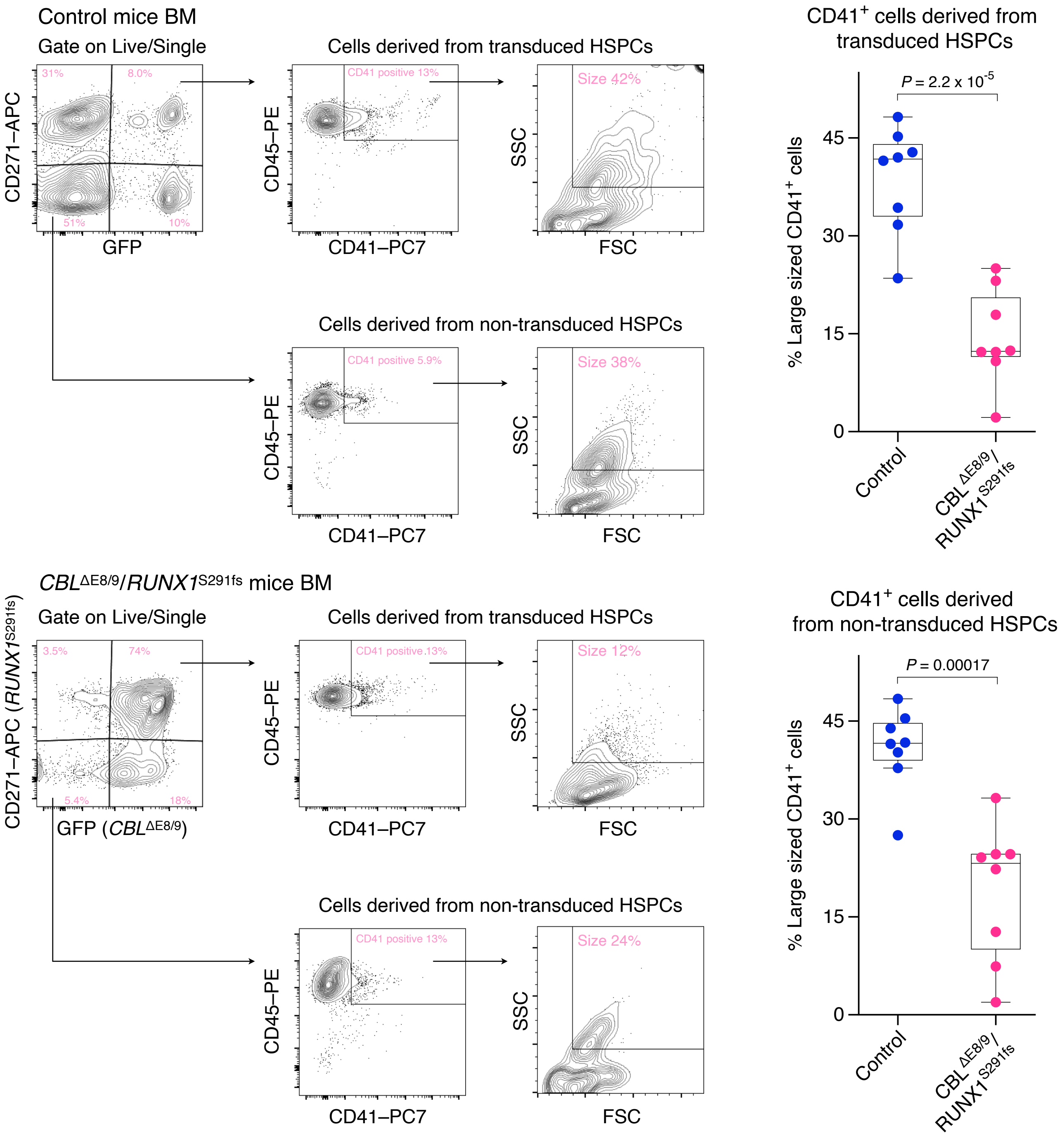

Supplementary Fig. 3

Mutant clones can affect the size of MK derived from wild-type clones. The gating strategy to analyze the size of CD41^{+} cells in BM from the indicated mice (left). Quantified data from CD41^{+} cells derived from transduced HSPCs (upper right) and non-transduced HSPCs (lower right) are shown (n = 8). P-values were calculated using the Student's two-tailed t-test.

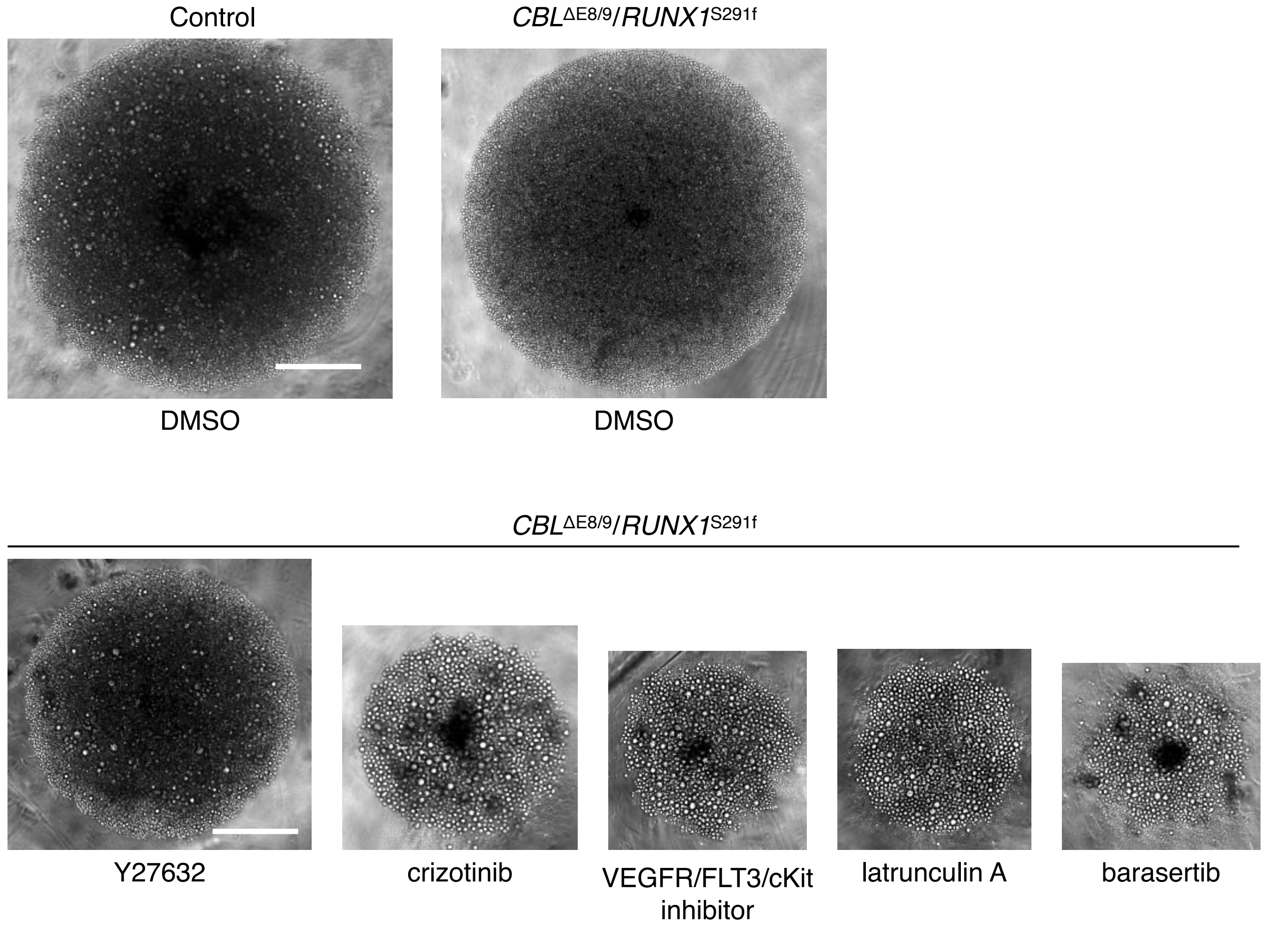

Supplementary Fig. 4

Identification of compounds that promote MK maturation. *Ex vivo* cultured HSPCs from an MDS model mouse were treated with each compound in a chemical collection for 4 days and then imaged. The data are from a single experiment. The images show that the compounds seem to increase the size of cells. Scale bar, 400 μm.

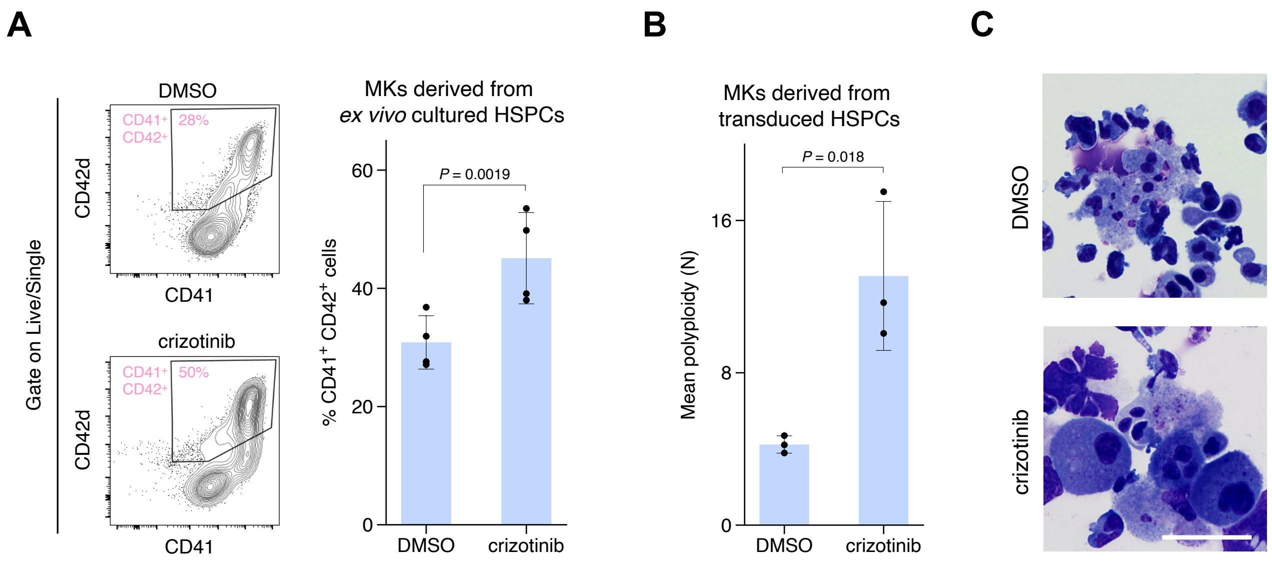

Supplementary Fig. 5

Effects of crizotinib on phenotypes of MKs derived from *ex vivo* cultured HSPCs from MDS model mice and HSPCs transduced with *CBL*$^{\Delta E8/9}$/*RUNX1*^{S291fs}. (**A**) Effect of crizotinib on the CD41 and CD42 expression in *ex vivo* cultured HSPCs from MDS mice. The data represent the mean ± s.d. of four independent experiments. (**B**) Effect of crizotinib on the ploidy of MKs derived from *CBL*$^{\Delta E8/9}$/*RUNX1*^{S291fs}–transduced HSPCs. The data represent the mean ± s.d. of three independent experiments. (**C**) Effect of crizotinib on the morphology of *CBL*$^{\Delta E8/9}$/*RUNX1*^{S291fs}–transduced HSPCs. Scale bar, 50 μm. *P*-values were calculated using the Student's two-tailed *t*-test (A, B)

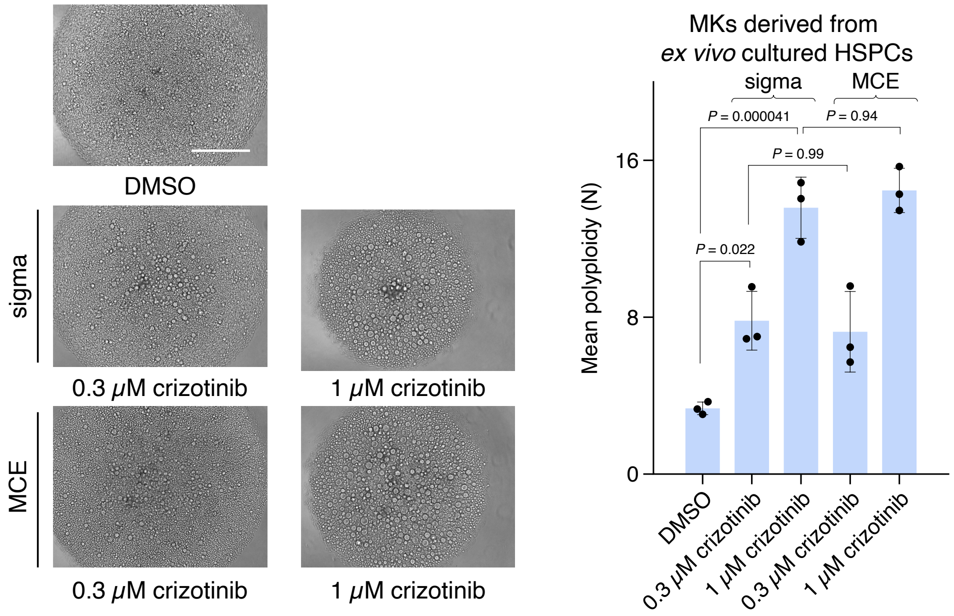

Supplementary Fig. 6

Crizotinib has bioactivity regardless of the chemical supplier. *Ex vivo* cultured HPSCs derived from an MDS mouse were treated with crizotinib purchased from Sigma or MedChemExpress (MCE) for 2 days, and the incubated for a further 2 days. The cells were imaged (left) and subjected to ploidy analysis (right). The data represent the mean ± s.d. of three independent experiments. *P*-values were calculated by one-way ANOVA with Tukey's multiple comparison test. Scale bar, 400 µm.

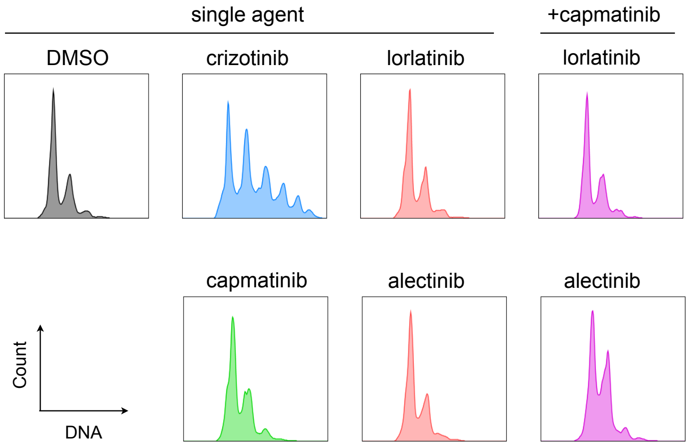

Supplementary Fig. 7

Crizotinib induces MK polyploidization by the inhibition of molecules other than ALK/ROS1/c-MET. Effects of inhibitors against the primary targets of crizotinib on the ploidy of MKs derived from *ex vivo* cultured HSPCs from MDS model mice. The concentration of each compound was 1 µM. Data are from a single experiment that was representative of two independent experiments.

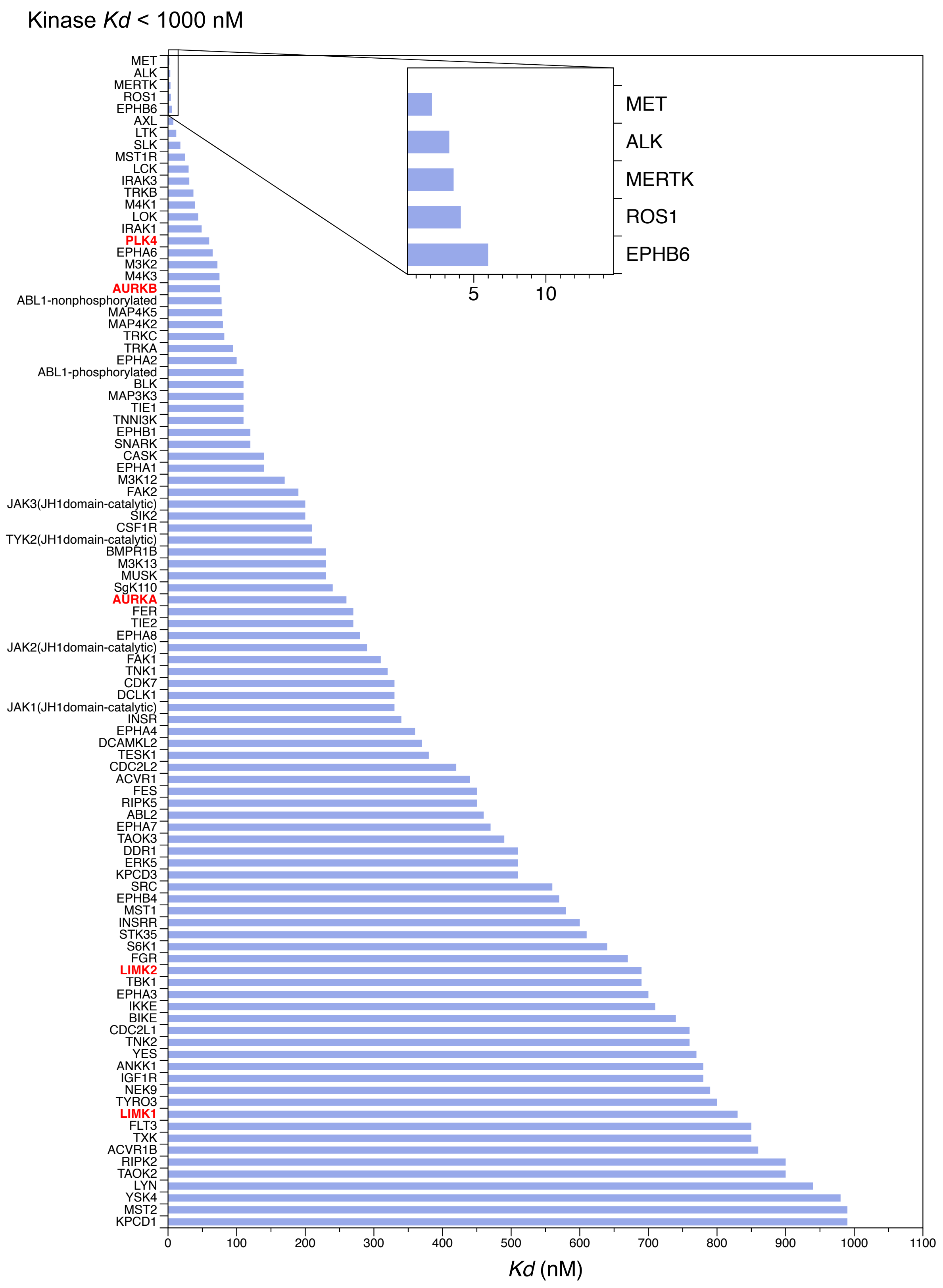

Supplementary Fig. 8

List of kinases to which crizotinib binds with a *Kd* value < 1000 nM, generated using data from crizotinib KINOMEscan (HMS LINCS Database, HMS Dataset ID: 20200). Gene names highlighted in red are cell cycle regulators and cytoskeletal organizers.

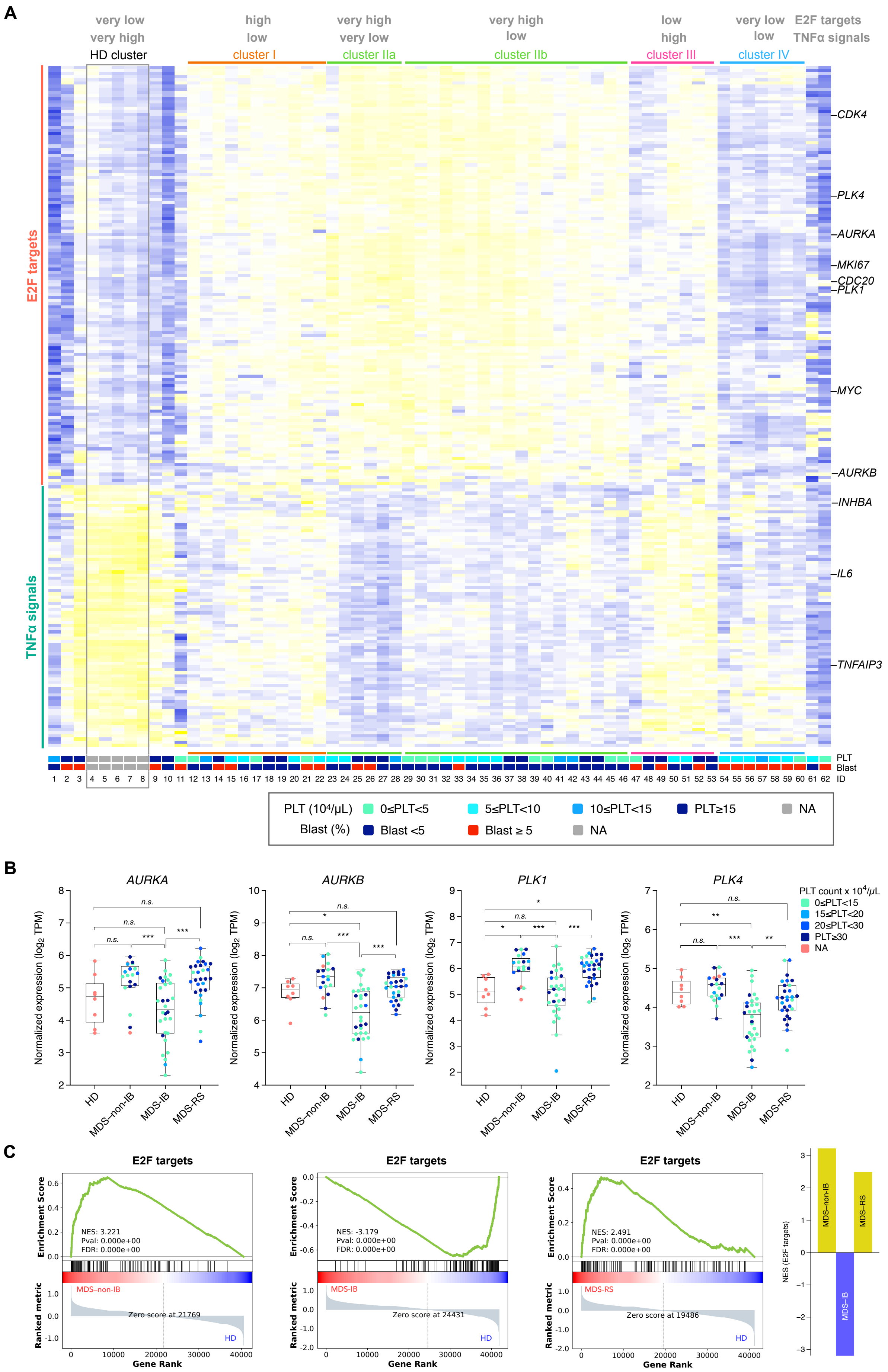
A
very low
very high
HD cluster
high
low
cluster I
very high
very low
cluster IIa
very high
low
cluster IIb
low
high
cluster III
very low
low
cluster IV
E2F targets
TNFα signals
CDK4
PLK4
AURKA
MKI67
CDC20
PLK1
MYC
AURKB
INHBA
IL6
TNFAIP3
PLT
Blast
ID
PLT (10⁴/μL) 0≤PLT<5 5≤PLT<10 10≤PLT<15 PLT≥15 NA
Blast (%) Blast <5 Blast ≥ 5 NA
B
AURKA
AURKB
PLK1
PLK4
Normalized expression (log2 TPM)
HD
MDS-non-IB
MDS-IB
MDS-RS
PLT count x 10⁴/μL
0≤PLT<15
15≤PLT<20
20≤PLT<30
PLT≥30
NA
C
E2F targets
Enrichment Score
Ranked metric
Gene Rank
NES: 3.221
Pval: 0.000e+00
FDR: 0.000e+00
MDS-non-IB
Zero score at 21769
HD
NES: -3.179
Pval: 0.000e+00
FDR: 0.000e+00
MDS-IB
Zero score at 24431
NES: 2.491
Pval: 0.000e+00
FDR: 0.000e+00
MDS-RS
Zero score at 19486
NES (E2F targets)

Supplementary Fig. 9

Gene expression analyses in CD34$^+$ cells derived from MDS patients. (**A**) Hierarchical clustering analysis using gene expressions of E2F targets and TNFα signals (provided in Supplementary Table 3) in BM CD34$^+$ cells from patients with MDS (n = 57) and HD (n = 5). PLT, platelet count. (**B**) Gene expression analysis of *PLK1*, *AURKA*, *AURKB*, and *PLK4* in BM CD34$^+$ cells from MDS–non-IB patients (n = 19), MDS-IB patients (n = 30), MDS-RS patients (n = 29), and HD (n = 8) using GSE114922 dataset. TPM, transcripts per million. (**C**) GSEA plots of E2F targets in BM CD34$^+$ cells from MDS patients (MDS–non-IB, MDS-IB, and MDS-RS) vs. those from HD (GSE114922). *P*-values were calculated using one-way ANOVA with Tukey's multiple comparison test (B). *n.s.*, not significant; *, $P < 0.05$; **, $P < 0.01$; ***, $P < 0.001$.

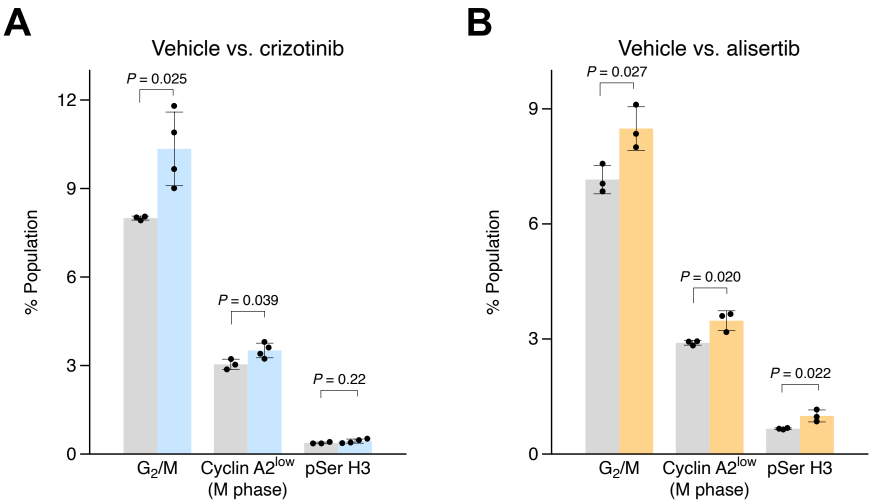

Supplementary Fig. 10

***In vivo* pharmacodynamic assays for crizotinib and alisertib.** C57BL/6 mice were treated once daily for 5 days with either vehicle (n = 3) or 100 mg/kg crizotinib (n = 4) (**A**) or vehicle (n = 3) or 30 mg/kg alisertib (n = 4) (**B**). BM cells were collected from each mouse 2 hours after the final administration, fixed, permeabilized, and stained with DAPI and either anti-Cyclin A2 or anti-pSer H3 antibodies. Samples were analyzed by intracellular flow cytometry. The gating strategies are included in Supplementary Data 4. *P*-values were calculated using the Student's two-tailed *t*-test (A, B).

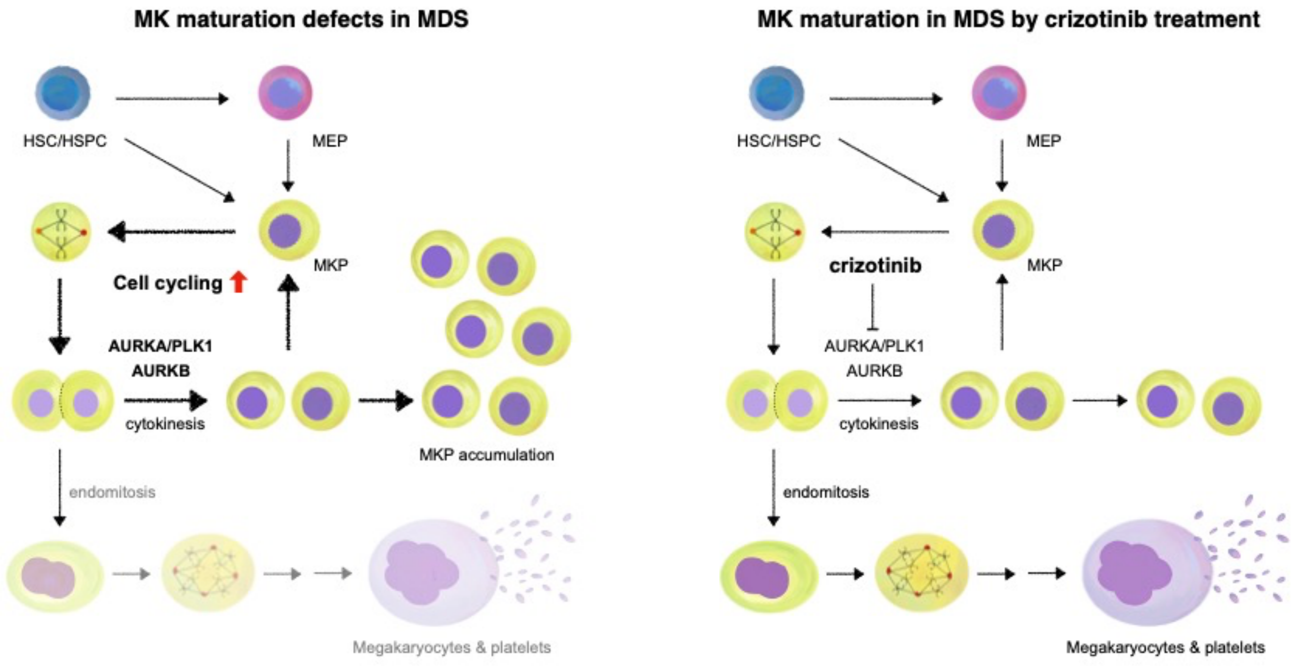

Supplementary Fig. 11

Schematic illustration of the model of MK maturation promoted by crizotinib. Left: in MDS with thrombocytopenia (especially unassociated with blasts), megakaryocyte progenitors progress cytokinesis/cell cycling mediated by activation of the cell cycling program and avoid endomitosis. Right: crizotinib inhibits Aurora kinases, decelerating cell cycling and redirecting cells from cytokinesis toward endomitosis, thereby promoting MK maturation. HSC, hematopoietic stem cell.

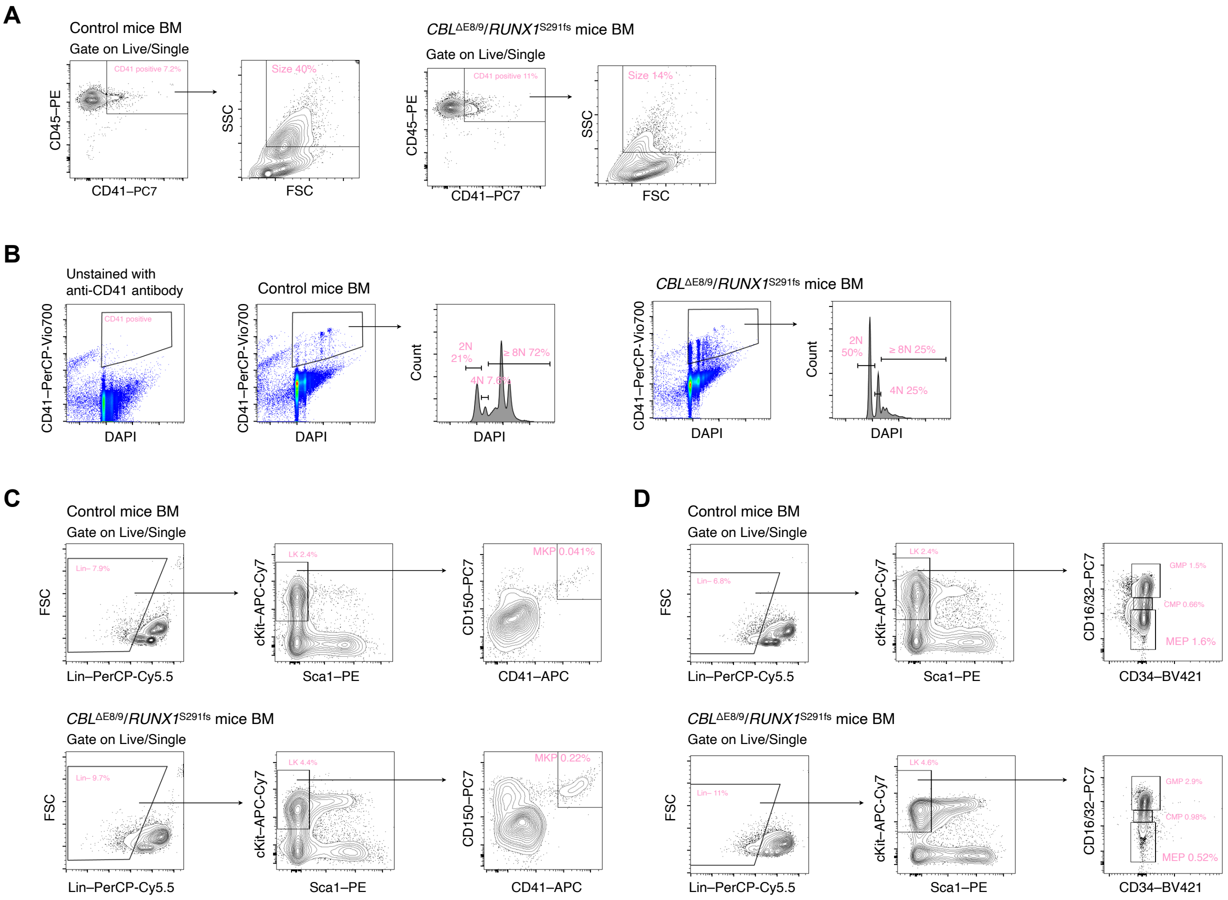

Supplementary Data 1

The gating strategies for Fig. 1B (**A**), Fig. 1C (**B**), Fig. 1E (**C**), and Fig.1F (**D**)

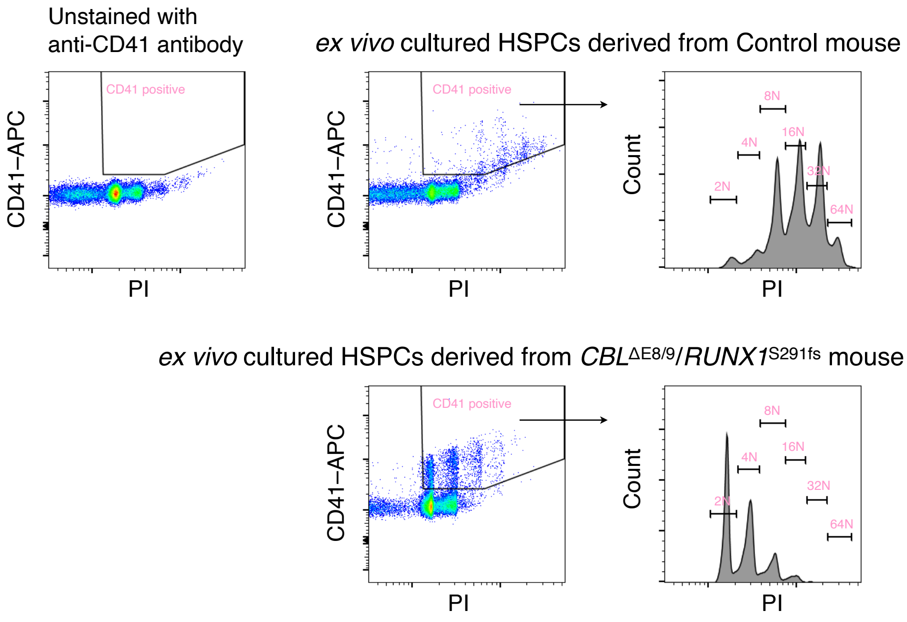

Supplementary Data 2

The gating strategies for Fig. 2B and Supplementary Fig. 2B.

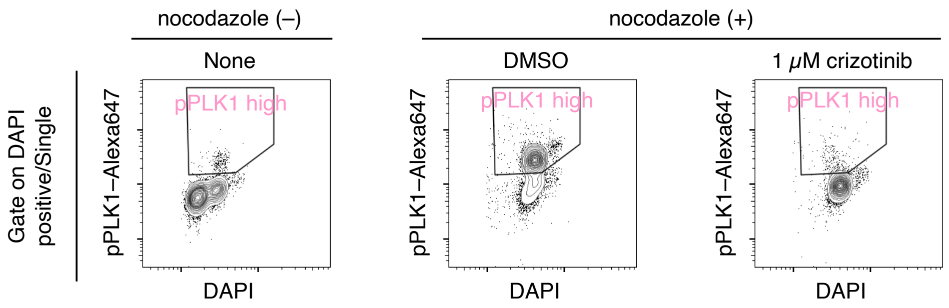

Supplementary Data 3

The gating strategy for Fig. 4C.

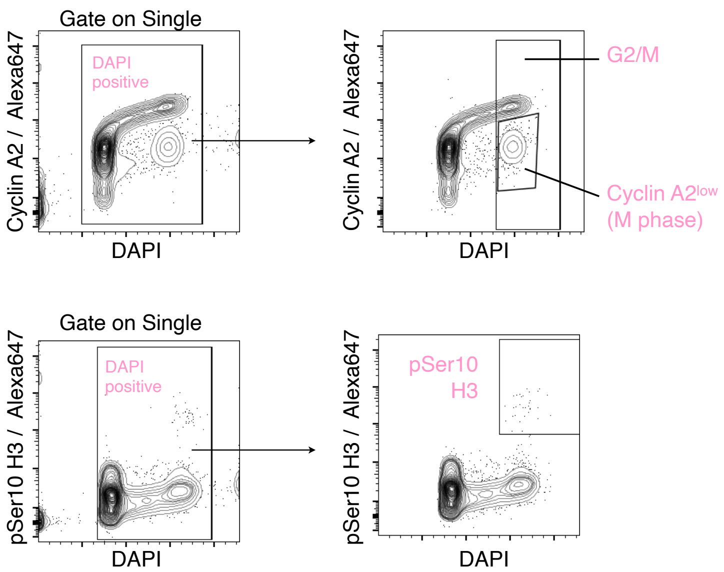

Supplementary Data 4

The gating strategies for Supplementary Fig. 10A, B.

Legends for Supplementary Tables

Supplementary Table 1

Top 100 gene sets positively enriched in BM CD34$^+$ cells from MDS patients with thrombocytopenia vs. those from HD are listed with NES, *p*-value, *q*-value, and count. "Count" indicates the number of genes contributing to the enrichment score. Gene sets highlighted in red are related to cell cycle progression.

Supplementary Table 2

Top 100 gene sets negatively enriched in BM CD34$^+$ cells from MDS patients with thrombocytopenia vs. those from HD are listed with NES, *p*-value, *q*-value, and count. "Count" indicates the number of genes contributing to the enrichment score.

Supplementary Table 3

The gene lists of E2F targets and TNFα signals. *AURKA, AURKB, PLK1*, and *PLK4* are highlighted in bold. These gene sets consist of MDS-leading edge subsets in HALLMARK_E2F_TARGETS and HALLMARK_TNFA_SIGNALING_VIA_NFKB.

Supplementary Table 4

List of samples from patients used in Figs. 4D, 4E, 5A and Supplementary Fig. 9A. All participants were Asian. "1" in the columns of (Fig. 4D, E), (SFig. 9A), and (Fig. 5A) indicates that the sample was used for the experiment. WBC, white blood cell. Hb, hemoglobin. PLT, platelet.

Supplementary Table 5

GSEA of BM CD34$^+$ cells from MDS–non-IB patients vs. those from HD (GSE114922) using Hallmark gene sets, E2F targets, and TNFα signals. All the gene sets tested are listed with NES, *p*-value, *q*-value, and count. "Count" indicates the number of genes contributing to the enrichment score.
